# Reference Values for Biochemical Analytes in An Iranian Population: The Tehran Lipid and Glucose Study

**DOI:** 10.5812/ijem-167774

**Published:** 2026-04-28

**Authors:** Shayan Sadrinasab, Sajad Jeddi, Fereidoun Azizi, Asghar Ghasemi

**Affiliations:** 1 Endocrine Physiology Research Center, Research Institute for Endocrine Molecular Biology, Research Institute for Endocrine Sciences, Shahid Beheshti University of Medical Sciences, Tehran, Iran; 2 Endocrine Research Center, Research Institute for Endocrine Disorders, Research Institute for Endocrine Sciences, Shahid Beheshti University of Medical Sciences, Tehran, Iran

**Keywords:** Biochemical Parameters, Clinical Diagnosis, Healthy Population, Iranian Healthy Adults, Reference Values, Pediatric, TLGS

## Abstract

**Context:**

Reference values (RVs) are essential tools for medical decision-making, used to interpret an individual’s health status and playing a crucial role in patient care. In addition to age, sex, and inherent biological variability, multiple determinants, including ethnicity, genetic background, dietary habits, environmental factors, and lifestyle, which vary across different populations, play a significant role in the establishment of RVs. Thus, each population requires its own RVs to ensure their effectiveness.

**Evidence Acquisition:**

In this review, we summarize RVs reported in the Tehran Lipid and Glucose Study (TLGS) between 2010 and 2026, covering 20 analytes measured in apparently healthy participants, including 16 in adults and 4 in pediatrics. Relevant TLGS publications were identified, and their findings were compared with corresponding international data.

**Results:**

RVs were provided for 20 parameters, classified into three categories: (1) Glucose, insulin, insulin resistance/sensitivity indices, and lipid profile; (2) thyroid function tests [thyroid-stimulating hormone (TSH) and free thyroxine (T4)] and autoimmunity [thyroid peroxidase antibody (TPO-Ab)]; (3) other variables, including creatinine and minerals (serum zinc and magnesium), and serum nitric oxide metabolites.

**Conclusions:**

The RVs determined by the TLGS provide a population-specific framework for the Iranian population and may serve as a more accurate tool for classification and clinical interpretation than common global RVs. These data emphasize the importance of developing localized RVs to enhance diagnostic precision and improve population health outcomes.

## 1. Context

The ultimate aim of biomedical science is to promote health, enhance patient care, and provide cost-effective healthcare services (1). Because health is a relative concept, tools are needed to distinguish healthy from unhealthy subjects (2). Reference values (RVs) are among the most widely used medical decision-making tools for interpreting a subject’s health status and play a crucial role in patient care (3). Although adopting common RVs may seem preferable at first glance, the practical application of this simple concept is challenging and does not justify such an approach (4, 5).

In addition to age, sex, and inherent biological variability, multiple determinants, including ethnicity, genetic background, dietary habits, environmental factors, and lifestyle, vary across populations and play significant roles in establishing RVs (6-8). Thus, each population requires its own RVs to ensure their effectiveness (9).

Reference values are derived from subject-based or population-based studies. Small, nonrepresentative sample sizes limit the generalizability of subject-based RVs, which may not accurately reflect the broader population (10-12). In contrast, population-based RVs, derived from large numbers of healthy individuals from the general population, yield more valid and broadly applicable RVs (13). Additionally, their large sample sizes increase statistical power and enable detailed subgroup analyses (14).

The use of inappropriate RVs can lead to misclassification, resulting in false-positive (15) or false-negative (16) results. False-positive results impose unnecessary psychological stress, diagnostic examinations, follow-ups, and medical care, all of which increase healthcare costs (17). Conversely, false-negative results can delay disease diagnosis, worsen prognosis, and ultimately increase morbidity (16). The Tehran Lipid and Glucose Study (TLGS) is a population-based prospective cohort study initiated in 1999 in Tehran, Iran, aimed at preventing noncommunicable diseases. In this study, a multistage stratified cluster sampling approach was used to recruit more than 15000 participants aged 3 years and older (18, 19). Data collected in the TLGS have paved the way for establishing population-based RVs for a broad array of measurements. The objective of this study was to summarize previously reported RVs for biochemical analytes in the TLGS, which can serve as decision-making tools for the Iranian population.

## 2. Adult Reference Values Determined in the Tehran Lipid and Glucose Study

As shown in Tables 1 - 3, RVs for 16 parameters in healthy adult subjects have been reported in TLGS data from 2010 to 2019. These parameters can be divided into three categories: (1) Glucose (20), insulin (21), insulin resistance/sensitivity indices (21), and lipid profile (22); (2) thyroid function tests [thyroid-stimulating hormone (TSH) and free thyroxine (T4) (23)] and autoimmunity [thyroid peroxidase antibody (TPO-Ab) (24)]; and (3) other variables, including creatinine, as the most commonly used renal function test (25), minerals [serum zinc and magnesium (9, 26)], and serum nitric oxide metabolites (27). All reported studies included both male and female subjects, with an age range of 20 - 93 years. In addition, standard methods for determining RVs (ie, parametric and nonparametric methods) according to guidelines presented by the Clinical and Laboratory Standards Institute (CLSI) (28), the International Federation of Clinical Chemistry and Laboratory Medicine (IFCC) (29), and the National Academy of Clinical Biochemistry (NACB) (30) were used. For conversion between different measurement units, readers can find conversion factors elsewhere (31, 32).

### 2.1. Glucose, Insulin, Insulin Resistance/Sensitivity Indices, and Lipid Profile

The RV for serum glucose concentration in the TLGS was found to be 70 - 100 mg/dL in both men and women (20). This range is consistent with the American Diabetes Association (ADA) criteria for defining normal fasting glucose [70 - 100 mg/dL (33)], values used at Massachusetts General Hospital (34), and values reported from Nordic countries (35) and Japan (36). Reference values for serum insulin concentration in the TLGS [2 - 12 μU/mL in both men and women (21)] were similar to those reported in the adult French population (37, 38). The lower limit of insulin RVs in our population (~2 μU/mL) is consistent with other reports (34, 37, 39); however, the upper limit of fasting insulin RVs in our population (~12 μU/mL) was lower than that reported in China [16 μU/mL (39)] or at Massachusetts General Hospital [20 μU/mL (39)]. A likely explanation for this difference is the method used to measure insulin (40). It has been shown that circulating insulin levels measured using different methods can differ by up to 2-fold, a point that both clinicians and laboratorians should consider (41). Overall, common RVs can be used for fasting glucose, but not for fasting insulin, across different populations. Reference values for insulin sensitivity/resistance indices [homeostasis model assessment of insulin resistance (HOMA-IR) and quantitative insulin sensitivity check index (QUICKI)] have also been reported in the TLGS (Table 1). Although these parameters are not currently used in clinics for diagnosis or treatment, accumulating evidence indicates their future applicability, particularly in relation to cardiovascular disease in type 2 diabetes (42).

**Table 1.** Reference Values for Fasting Serum Glucose, Insulin, and Lipid Profile in Iranian Healthy Adults: Tehran Lipid and Glucose Study ^a^

Analyte	Year	n	Sex (M/F)	Age, y	Method for Determining RVs	Measurement Method	RVs, Males	RVs, Females	Unit	Intra-assay CV, %	Inter-assay CV, %	Ref
**Glucose**	2011	962	404/558	20 - 78	IFCC/nonparametric and robust methods	Enzymatic colorimetric (glucose oxidase)	72 - 103	68 - 101	mg/dL	2.2	2.2	(20)
**Insulin**	2014	309	123/181	24 - 83	CLSI/IFCC/nonparametric and robust methods	Electrochemiluminescence immunoassay (ECLIA)	1.6 - 11.4	2.3 - 12.0	μU/mL	1.2	3.5	(21)
**HOMA1-IR**	2014	275	108/167	24 - 83	—	HOMA equation	0.61 - 2.38	0.61 - 2.68	—	—	—	—
**HOMA2-IR**	2014	275	108/167	24 - 83	—	HOMA2 calculator	0.29 - 1.58	0.40 - 1.80	—	—	—	—
**QUICKI**	2014	275	108/167	24 - 83	—	QUICKI equation	0.22 - 0.42	0.33 - 0.42	—	—	—	—
**TC**	2019	1143	546/597	20 - 93	—	Enzymatic colorimetric (cholesteryl ester hydrolase)	121 - 261	118 - 236	mg/dL	3.0	3.0	(22)
**LDL-C**	2019	1143	544/599	20 - 93	—	Friedewald equation	54 - 175	50 - 161	mg/dL	—	—	—
**HDL-C**	2019	1147	548/599	20 - 93	—	Enzymatic colorimetric (lipoprotein lipase)	31 - 72	36 - 84	mg/dL	4.6	4.6	—
**TG**	2019	1145	548/597	20 - 93	—	Enzymatic colorimetric (lipoprotein lipase)	47 - 301	38 - 184	mg/dL	2.3	2.3	—

Abbreviations: CLSI, clinical and laboratory standards institute; CV, coefficient of variation; ECLIA, electrochemiluminescence immunoassay; F, female; HDL-C, high-density lipoprotein cholesterol; HOMA-IR, homeostasis model assessment of insulin resistance; IFCC, International Federation of Clinical Chemistry and Laboratory Medicine; LDL-C, low-density lipoprotein cholesterol; M, male; QUICKI, quantitative insulin sensitivity check index; TC, total cholesterol; TG, triglycerides.

^a^ To convert glucose values from mg/dL to mmol/L, multiply by 0.05551. To convert TG values from mg/dL to mmol/L, multiply by 0.01129. To convert TC, LDL-C, and HDL-C values from mg/dL to mmol/L, multiply by 0.02586. To convert insulin values from μU/mL to pmol/L, multiply by 6.945 (31, 32).

Reference values for the lipid profile determined in healthy TLGS subjects (Table 1) differed between men and women, with higher upper limits for total cholesterol (TC), triglycerides (TG), and low-density lipoprotein cholesterol (LDL-C), as well as a lower lower-limit value for high-density lipoprotein cholesterol (HDL-C), observed in men compared with women (22). This finding is consistent with results from Canada (43), Finland (44), India (45, 46), and Turkey (47). The difference in HDL-C values is related to sex hormones, because estrogen increases and testosterone decreases serum HDL-C (48, 49). It should be noted that RVs for lipid profiles vary widely across populations (43, 45-47). This variation has been attributed to ethnicity, dietary habits, genetic background, environmental factors, and lifestyle (44, 45, 50). In addition, LDL-C may be measured directly or calculated using the original Friedewald formula (LDL-C = TC - HDL-C - TG/5) (51). However, it has been shown that a modified version of the Friedewald formula should be used across different populations; this modified version has also been reported in the TLGS (LDL-C = TC - HDL-C - TG/4) (52).

### 2.2. Thyroid Function Tests and Autoimmunity

The RVs for thyroid-related parameters reported in this review were derived from the Tehran Thyroid Study (TTS), an ancillary study conducted within the framework of the TLGS (53). Population-based RVs for serum TSH (0.31 - 4.14 mIU/L in men and 0.32 - 5.63 mIU/L in women) and free T4 (0.95 - 1.60 ng/dL in men and 0.89 - 1.50 ng/dL in women) in TPO-Ab-negative healthy Iranian subjects were reported in the TLGS for the first time (23) (Table 2). The most commonly used TSH RVs are 0.5 - 5 mIU/L, and the NACB recommends reducing this range to 0.4 - 2.5 mIU/L (54). Despite some minor differences across populations (55, 56), RVs for both serum TSH and free T4 obtained in the TLGS are consistent with those reported in Denmark (57), India (58), and Spain (59). It should be noted that the measurement method, iodine status, and exclusion criteria used may affect RVs. For example, some studies did not exclude TPO-Ab-positive subjects (60, 61); however, these subjects were excluded in the TLGS in accordance with NACB standards to obtain disease-free reference populations (54).

**Table 2.** Reference Values for Serum TSH, Free T4, and TPO-Ab in Iranian Healthy Adults: Tehran Thyroid Study, an Ancillary Study of the Tehran Lipid and Glucose Study ^a^

Analyte	Year	n	Sex (M/F)	Age, y	Method for Determining RVs	Measurement Method	RVs, Males	RVs, Females	Unit	Intra-assay CV, %	Inter-assay CV, %	Ref.
**TSH**	2013	2199	953/1246	≥ 20	NACB guidelines	ECLIA	0.31 - 4.14	0.32 - 5.63	mU/L	1.4	4.6	(23)
**Free T4**	2013	2199	953/1246	≥ 20	NACB guidelines	ECLIA	0.95 - 1.60	0.89 - 1.50	ng/dL	1.3	3.3	—
**TPO-Ab**	2015	2823	1081/1742	20 - 80	NACB guidelines	ELISA	1.50 - 32.8	1.55 - 35.0	IU/mL	3.9	4.7	(24)

Abbreviations: CV, coefficient of variation; ECLIA, electrochemiluminescence immunoassay; ELISA, enzyme-linked immunosorbent assay; F, female; M, male; NACB, National Academy of Clinical Biochemistry; T4, thyroxine; TPO-Ab, thyroid peroxidase antibody; TSH, thyroid-stimulating hormone.

^a^ To convert free T4 values from ng/dL to pmol/L, multiply by 12.87 (31).

The RVs for serum TPO-Ab in adults in the TLGS were 1.50 - 32.8 IU/mL in men and 1.55 - 35.0 IU/mL in women (24) (Table 2). The upper limit of TPO-Ab found in the TLGS is consistent with the NACB standard (ie, 30 IU/mL) (54). This value is also consistent with those reported from Australia (62) and Italy (63). However, lower upper limits have also been reported in other populations [eg, 16 IU/mL in Japan (64) and 15 IU/mL in Danish Caucasians (65)]. In addition, TPO-Ab values are affected by measurement method, iodine intake status [because both iodine excess and deficiency increase TPO-Ab positivity (66)], genetics [certain human leukocyte antigen (HLA) types are associated with higher TPO-Ab levels (67)], age, and sex [for example, TPO-Ab increased with age in females (68)]. It has been shown that the upper limits of TPO-Ab RVs range from 6 to 29 IU/mL in men and from 7 to 29 IU/mL in women, depending on the method used (63), indicating a 4- to 5-fold difference. This information should be considered when interpreting serum TPO-Ab values.

### 2.3. Creatinine, Magnesium, Zinc, and Nitric Oxide Metabolites

In the TLGS, RVs for serum creatinine [0.53 - 1.11 mg/dL for men and 0.42 - 0.77 mg/dL for women (25)] were determined using the compensated Jaffe method (Table 3). These values are approximately 0.32 mg/dL lower than those measured using the conventional Jaffe method, a factor that should be considered when interpreting serum creatinine RVs (see the next section). Upper limits of creatinine RVs, which are clinically more relevant than lower ranges (69), in our population were close to those reported from China (70) and Kenya (71) but lower than those observed in European populations (72). Lower muscle mass in Asian populations may partially explain this discrepancy (73).

**Table 3.** Reference Values for Fasting Serum Creatinine, Magnesium, Zinc, and Nitric Oxide Metabolites in Iranian Healthy Adults: Tehran Lipid and Glucose Study ^a^

Analyte	Year	n	Sex (M/F)	Age, y	Method for Determining RVs	Measurement Method	RVs, Males	RVs, Females	Unit	Intra-assay CV, %	Inter-assay CV, %	Ref.
**Creatinine**	2014	5247	2792/2455	20 - 88	CLSI/IFCC/nonparametric method	Photometric Jaffe	0.53 - 1.11	0.42 - 0.77	mg/dL	2.2	4.1	(25)
**Magnesium**	2010	491	233/258	20 - 50	IFCC/parametric and robust methods	Atomic absorption spectrometry	1.8 - 2.5	1.8 - 2.5	mg/dL	0.84	2.5	(26)
**Zinc**	2012	2632	1119/1513	20 - 90	IFCC/nonparametric method	Atomic absorption spectrometry	63 - 206	58 - 195	µg/dL	3.7	5.4	(9)
**Nitric oxide metabolites**	2010	687	244/442	≥ 20	IFCC/parametric and robust methods	Spectrophotometric (Griess assay)	11 - 76	10 – 66 ^b^	µmol/L	4.2	4.9	(27)

Abbreviations: CLSI, Clinical and Laboratory Standards Institute; CV, coefficient of variation; F, female; IFCC, International Federation of Clinical Chemistry and Laboratory Medicine; M, male.

^a^ To convert creatinine values from mg/dL to mmol/L, multiply by 88.4. To convert zinc values from μg/dL to μmol/L, multiply by 0.153. To convert magnesium values from mg/dL to mmol/L, multiply by 0.411 (31).

^b^ Women with menopause were not included.

As shown in Table 3, RVs for serum magnesium [1.8 - 2.5 mg/dL in both sexes (26)] were almost similar to those reported in Germany (74) and the United Kingdom (75), and minor differences may be attributed to ethnic variation (74, 76) and the more restrictive selection criteria in our study, which eliminated most magnesium metabolism-related problems (75). Reference values for serum zinc concentrations (Table 3) in the TLGS were 60 - 200 µg/dL for both sexes (9). Typical RVs currently used by most studies are 60 - 120 (77) or 70 - 120 μg/dL (78). The lower limits of serum zinc RVs in our population are consistent with those reported in Germany (79) and Kuwait (80), and our upper limits are consistent with those reported in the Canary Islands (81) and northeast Thailand (82). However, serum zinc RVs vary widely across populations (80, 83, 84). Although differences in dietary habits may partly explain these differences, this issue highlights the need to use local RVs (85) and warrants further investigation.

Adult RVs for serum nitric oxide metabolites in the TLGS (10 - 70 µM in both sexes, using the Griess assay) are the first population-based adult RVs for serum nitric oxide metabolites (27) (Table 3). These values support those obtained in small-sample studies conducted in healthy subjects using the Griess assay (8 - 75 µM) (86-89), high-performance liquid chromatography (HPLC) method (10 - 80 µM), and gas chromatography-mass spectrometry method (20 - 85 µM), which is the most accurate quantitative method for measuring nitric oxide. Nitric oxide metabolites are not currently measured routinely in clinics (89); however, accumulating evidence indicates that they may be used in the future, for instance, in septic shock, in which very high nitric oxide production is a major culprit in shock-induced hypotension that is resistant to available antihypertensive treatments (90).

## 3. Pediatric Age-Group Reference Values Determined in the Tehran Lipid and Glucose Study

As shown in Table 4, RVs for serum creatinine, magnesium, zinc, and nitric oxide metabolites have been determined in pediatric populations in the TLGS. These studies were conducted between 2010 and 2015 and included children and adolescents aged 3 - 19 years. Among these values, RVs for serum creatinine in the TLGS are consistent with those reported in other countries, including the United Kingdom (91) and Canada (92). For creatinine, we used two methods (the conventional Jaffe method and compensated Jaffe method), which provided two sets of RVs [conventional method: 0.6 - 1.2 mg/dL for boys and 0.6 - 1.0 mg/dL for girls; compensated method: 0.23 - 0.86 mg/dL for boys and 0.23 - 0.65 mg/dL for girls (93)]. The higher values observed with the conventional Jaffe method are due to assay interference from noncreatinine chromogens, which can be avoided by subtracting a constant value (0.35 mg/dL) from the results (94), yielding more specific and accurate creatinine measurements in children. Therefore, both laboratory staff and clinicians should be aware of the measurement method when reporting or interpreting RVs for serum creatinine in pediatric populations.

**Table 4.** Reference Values for Fasting Serum Analytes in Iranian Healthy Pediatric Age-Group: Tehran Lipid and Glucose Study ^a^

Analyte	Year	n	Sex (M/F)	Age, y	Method for Determining RVs	Measurement Method	RVs, Males	RVs, Females	Unit	Intra-assay CV, %	Inter-assay CV, %	Ref.
**Creatinine**	2015	1502	778/724	3 - 18	CLSI/IFCC guidelines/nonparametric method	Photometric conventional Jaffe	0.60 - 1.20	0.60 - 1.00	mg/dL	3.9	4.7	(93)
**Creatinine**	2015	1502	778/724	3 - 18	CLSI/IFCC guidelines/nonparametric method	Photometric compensated Jaffe	0.23 - 0.86	0.23 - 0.65	mg/dL	3.9	4.7	(93)
**Magnesium**	2010	146	64/82	3 - 19	IFCC/parametric and robust methods	Flame atomic absorption spectrometry	1.8 - 2.4	1.8 - 2.4	mg/dL	0.84	2.5	(95)
**Zinc**	2012	672	323/349	3 - 18	CLSI/IFCC/nonparametric method	Flame atomic absorption spectrometry	63 - 206	60 - 202	µg/dL	NR	NR	(94)
**Nitric oxide metabolites**	2010	401	189/212	4 - 19	IFCC/parametric and robust methods	Griess	14 - 69	11 - 66	µmol/L	4.2	4.9	(96)

Abbreviations: CLSI, Clinical and Laboratory Standards Institute; CV, coefficient of variation; F, female; IFCC, International Federation of Clinical Chemistry and Laboratory Medicine; M, male; NR, not reported.

^a^ To convert values from conventional units to SI units, see Table 3.

Pediatric RVs for serum magnesium [1.8 - 2.4 mg/dL for both sexes (95)] are consistent with those reported from other countries, including Romania (97), Croatia (98), and the United Kingdom (99). In addition, these values closely match those of healthy adults in the TLGS [1.8 - 2.5 mg/dL (26)], suggesting that a common RV for serum magnesium can be adopted. This is not the case for pediatric serum zinc RVs in our population [60 - 200 µg/dL in both sexes (94)]. A wide range in both lower limits (as low as 35 µg/dL) and upper limits (as high as 220 µg/dL) of serum zinc RVs has been reported across populations (79), underscoring the need to determine local RVs (85). These differences may be due to dietary habits and regional factors, such as environmental pollution and local agriculture (85).

To the best of our knowledge, population-based pediatric RVs for serum nitric oxide metabolites (10 - 70 µmol/L in both sexes) have been reported only in the TLGS (96). These values support those reported in other small-sample studies conducted in healthy children (100, 101).

In summary, pediatric RVs have not been extensively investigated in the TLGS population, possibly because of the difficulty of establishing exclusion criteria during steady-state growth in children. However, knowledge in this field is required to provide better health care for this specific population.

## 4. Quality Control

Quality control (QC) involves procedures to monitor and evaluate analytical methods and testing processes, aiming to detect and correct errors. It ensures that laboratory results are valid (accurate and fulfilling their claims) and thus suitable for subsequent applications (102, 103). Accuracy encompasses both trueness (closeness to the true value) and precision (minimal dispersion among repeated measurements) (102, 104). Trueness is the extent to which measurement results are free from systematic error or bias (mean of measurements - true value) (105, 106). In practice, laboratories use “controls” with prespecified values to determine trueness. Precision, the extent to which measurement results are free from random error (value of a single measurement - value of the mean of measurements), is a precondition for validity (107). In practice, precision is expressed as intra-assay and inter-assay coefficients of variation (CVs).

In the TLGS, a standard QC system was implemented to ensure the validity of the collected data (18, 108). The CVs for the analytes measured in the TLGS (Tables 1 - 4) are all within acceptable ranges, indicating the validity and precision of the assays employed (109). Based on standard laboratory practices, acceptable CVs are as follows: glucose, ≤ 2.4% (110); TC, ≤ 3% (111); TG, ≤ 5% (111); HDL-C, ≤ 4% (111); TSH, ≤ 5.6% (112); free T4, ≤ 3.8% (112); anti-TPO, ≤ 7% (113); creatinine, ≤ 8% (114); magnesium, ≤ 2.68% (115); and zinc, ≤ 7.55% (116). For insulin, CVs vary according to the measurement method and concentration range used; an intra-assay CV ≤ 10% and an inter-assay CV ≤ 13% are acceptable (41).

## 5. Conclusions and Future Perspectives

Reference values are essential tools in clinical decision-making and health assessment. Different genetic backgrounds, ethnicities, dietary habits, and environmental exposures warrant the establishment of RVs in each population. The TLGS, a large population-based cohort study, has provided a unique opportunity to define RVs for the Iranian population. Here, we summarized reported RVs for 16 parameters in adults and 4 parameters in pediatric populations, based on TLGS reports. Although common RVs can be adopted for some analytes (eg, serum glucose and magnesium), some RVs obtained in our population (eg, lipid profile and serum zinc) show substantial differences, warranting more attention from health decision-makers. In addition, RVs for other analytes (eg, liver function tests) need to be established. Finally, establishing RVs in pediatric populations is an understudied area in Iran. In conclusion, the RVs summarized from the TLGS in this study can be used by healthcare providers in Iran.

This study has several clinical and public health implications. First, for some analytes (eg, serum insulin), the measured value varies according to the measurement method, which should be considered when interpreting results. Second, for serum creatinine, values obtained by the compensated Jaffe method are lower than those obtained by the conventional Jaffe method, which requires careful consideration by clinicians. Finally, values for serum nitric oxide metabolites, although not routinely used in clinics, may be important for monitoring certain clinical conditions, particularly septic shock.

## Data Availability

The dataset presented in the study is available on request from the corresponding author during submission or after publication.
